# Men’s health: time for a new approach to policy and practice?

**DOI:** 10.7189/jogh.07.010306

**Published:** 2017-06

**Authors:** Peter Baker, Tim Shand

**Affiliations:** 1 Global Action on Men’s Health, London, UK; 2 Institute for Reproductive Health, Georgetown University, Washington DC, USA

The United Nation’s (UN) Sustainable Development Goal 3 on health and well–being contains important commitments to reducing by one third premature mortality from non–communicable diseases (NCDs), promoting mental health and well–being, strengthening the prevention and treatment of substance abuse, including narcotic drug abuse and harmful use of alcohol, and halving the number of global deaths and injuries from road traffic accidents. The Goal also aims to ensure universal access to sexual and reproductive health–care services, including for family planning, information and education, and to improve the implementation of the World Health Organization (WHO) Framework Convention on Tobacco Control.

All these commitments, if successfully implemented, would be particularly beneficial to the health of men and boys across the world; equally, they cannot be optimally realized without an approach that takes account of the specific health needs, social contexts and the related health practices of men and boys, and perceives addressing this area as a pathway to better well–being and equality for all. At present, such an approach is not reflected in policy and practice.

## MALE MORTALITY

WHO data shows that, globally in 2012, 52% of all deaths from NCDs were male. Males were more likely than females to die prematurely (under 70 years) from NCDs in almost every country (females were more likely to die prematurely from NCDs in just four countries). The proportion of premature NCD deaths in males was twice or more that in females in 11 countries, including Russia where 52% of male NCD deaths were premature compared to 24% of female NCD deaths.

The major risk factors for NCDs include unhealthy diets, tobacco use and the harmful use of alcohol. Men do worse than women in respect of all of these. Data from the Global Burden of Disease Study 2010 shows that, in that year, 55% of deaths from dietary risk factors were male as were 72% of deaths from tobacco smoking and 65% of deaths from alcohol. More males than females also died from environmental factors (unimproved water and sanitation, air pollution) and also drug use. There was a particularly large sex difference for deaths caused by occupational risks: 88% of deaths from this cause were male in 2010.

Males accounted for 82% of all homicide victims in 2012 and have estimated rates of homicide that are more than four times those of females (10.8 and 2.5, respectively, per 100 000), according to WHO data. Males were also almost twice as likely to die by suicide as women. In high–income countries, men were three times more likely to die by suicide.

Life expectancy data also highlights the health burden borne by men. Globally, male life expectancy at birth, at 68 years, lags five years behind female life expectancy and the global “gap” is predicted to increase over the next 15 years: by 2030, male life expectancy could well be seven years shorter than female life expectancy [1]. There is not a single country where male life expectancy exceeds female and there are currently 27 countries in the world with male life expectancy below 60.

## USE OF HEALTH SERVICES

The under–utilization of primary care services by men has also been identified as a problem in many countries. In Europe, infrequent use of, and late presentation to, such services has been associated with men experiencing higher levels of potentially preventable health conditions and having reduced treatment options [2]. This is particularly the case for mental health problems. Studies in sub–Saharan Africa have reported similar findings about men’s use of HIV services and also found that men are proportionally less likely to test for HIV and begin treatment regimes and more likely to die while on treatment [3–5]. Within the context of family planning, there has been little shift globally in men’s use of contraception over the last 20 years, with the burden of responsibility remaining firmly with women [6].

There are also significant structural barriers that inhibit men’s ability to self–care and to access services effectively. Many primary care services are available only at times when men are at work, for example [7]. It has been suggested that the feminine ambience of services also deters men who in any case view health as a predominantly female domain. Health awareness campaigns have often failed to engage men and have, deliberately or inadvertently, been aimed primarily at women [8].

## HEALTH LITERACY

Men tend to be less knowledgeable than women about specific diseases, risk factors and health in general. A recent study of weight, diet, physical activity and nutritional knowledge among university students in the USA found that men were more likely to be overweight or obese, more likely to consume red meat, fast food, sugar–sweetened beverages, wine and beer, and less likely to be knowledgeable about nutrition [9]. Other research has found that men are less likely to recognize that they are overweight and are less well–informed about the common symptoms of cancer.

## RISK–TAKING AND MASCULINITY

Men’s risk–taking behaviors and their under–use of health services are in large part linked to male role norms. These norms vary according to social and cultural contexts but also appear consistent across many countries in terms of health behaviors. In rural India, for example men’s use of tobacco is closely linked to their perception that a “real man” should be daring, courageous and confident and able to demonstrate his manliness by smoking [10]. A study of men in Russia suggested that heavy drinking of strong spirits “elevates or maintains a man’s status in working–class social groups by facilitating access to power associated with the hegemonic ideal of the real working man” [11]. Evidence shows that promoting positive models of manhood, such as caring and involved fatherhood, while concurrently addressing structural barriers, can improve men’s help and health–seeking behavior [12].

## TAKING ACCOUNT OF SEX AND GENDER

We do not argue that tackling men’s health is more important than addressing women’s health; in reality, there is not a binary choice to be made nor is this a zero sum game. In specific areas of health, women’s outcomes are worse than men’s. Moreover, in many countries, women are denied equal access to health services, and gender power dynamics mean they often lack autonomy in health–related decision–making. Women’s health problems are inextricably linked to many social, economic, legal, political and cultural forms of discrimination. It is therefore right that women should be regarded as a priority for action by global and national health organisations. As the data highlighted above shows, however, men also face a wide range of serious health problems which require a complementary approach.

The need to take account of sex and gender in relation to the health of both men and women is well–established in the literature. It is now a quarter of a century since England’s Chief Medical Officer included a path–breaking chapter on men’s health in his annual report on the state of the nation’s public health and emphasized the importance of paying greater attention to sex differences in disease susceptibility “to the benefit of men and women alike.” More recently, in a report on the social determinants of health for WHO Europe, Michael Marmot recommended that strategies should “respond to the different ways health and prevention and treatment services are experienced by men [and] women” and policies and interventions should be “responsive to gender” [13]. The head of WHO’s gender, equity and human rights group has also written about the importance of “capturing the different experiences of men and women” [14].

These insights have not yet been translated into action at the strategic level, however. An analysis of the policies and programmes of 11 major global health institutions, including WHO, found that they did not address the health needs of men [15]. The UN’s Global Strategy for Women’s, Children’s and Adolescents’ Health (2016–30) overlooks boys and world leaders at the 2016 G7 Ise–Shima Summit in Japan made important commitments to improving women’s health but did not mention men, or how they could be engaged to support improvements in women’s health. The flagship global strategy for increasing contraceptive uptake by an additional 120 million users, Family Planning 2020, includes only women as users and not men. Global health NGOs have shown insufficient interest in men as a specific group. Only four countries – Australia, Brazil, Iran and Ireland – have developed national men’s health policies. In most other countries, men’s health is not recognized as an issue of concern by governments or health providers.

## THE BENEFITS OF IMPROVED MEN’S HEALTH

As stated in the WHO Constitution, “the enjoyment of the highest attainable standard of health is one of the fundamental rights of every human being” [16]. Increasing men’s ability to lead healthy and fulfilling lives is an ethical imperative. Improving men’s health would not benefit just men, however. Improved sexual and reproductive health for men would have immediate and obvious benefits for women as well as men themselves. Lower male premature mortality and morbidity rates would reduce the burden on women and families who depend on men's incomes. Improved mental health and lower levels of alcohol consumption would help to reduce male violence toward partners and others.

Healthier men would reduce the economic costs of lost productivity and health treatments. Men’s premature mortality and morbidity has been estimated to cost the United States economy approximately US$ 479 billion annually [17] while the economic burden associated with smoking, excess weight, alcohol and physical inactivity in Canadian men is believed to be about CA$ 37 billion a year (US$ 28 billion). Retirement ages are rising internationally so it is increasingly important to enable men to remain economically active for longer.

## WHAT WORKS WITH MEN

There is a growing evidence base from around the world showing that well designed health interventions aimed at men can improve outcomes for themselves and others and transform harmful gender norms. The Football Fans in Training program in Scotland, now extended into other European countries as EuroFIT, shows that professional sport can be an effective medium for engaging men in lifestyle improvement programmes [18]. A study of the core elements that make for successful work with boys and men on mental health promotion, early intervention and stigma reduction found that the settings within which interventions take place need to be “male friendly” and culturally sensitive to the specific requirements of different groups of men and boys [19]. Interventions that aim to reshape male gender roles in ways that lead to more equitable relationships between women and men can reduce sexually transmitted infections and prevent intimate partner violence [20]. Easier–to–access primary care services could also reduce some of the barriers to service use experienced by men.

## NEXT STEPS

For progress to be made, we believe that global health organizations and national governments should, as part of a comprehensive approach to gender and health, address the health and well–being needs of men and boys in all relevant policies (eg, on obesity, cardiovascular disease and cancer) and through the introduction of specific men’s health policies. Educational programmes in schools and male–targeted health information can be used to encourage and support boys and men to take better care of their own health. Health practitioners must inform themselves about the psychosocial aspects of men’s health, as well as male–specific clinical issues, and medical training programmes should cover gender and other social determinants of health. Workplaces have a key role, in terms of not only reducing exposure to hazards but also providing a setting for health promotion.

**Figure:**
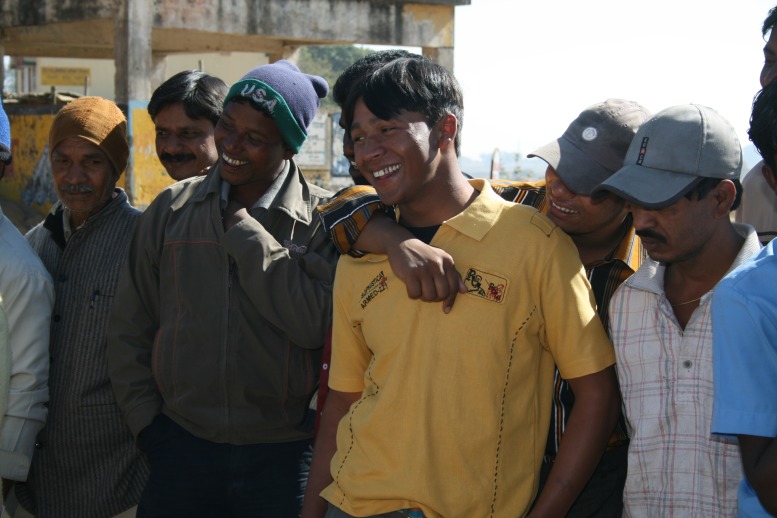
Photo: Courtesy of the Institute for Reproductive Health, Georgetown University, USA

It is essential for work with men to focus on those groups with the worst health, such as economically disadvantaged men, gay and bisexual men, men who are homeless, migrants or offenders, and men from specific racial and ethnic groups. It is important to recognize that most men want to enjoy good health and well–being and that their strengths and the “positive” aspects of masculinity (for example, a desire to provide for and protect one’s family) can be harnessed to help them achieve better outcomes. But in order to improve men’s health successfully, there must be a commensurate policy and programming response. Further research is also needed into how to influence men’s health behaviors and improve their use of primary care services.

One thing is clear: the Sustainable Development Goals and better health for all cannot be achieved if the many challenges currently facing men are left hiding in plain sight.
